# A method for the reconstruction of multifocal structured illumination microscopy data with high efficiency

**DOI:** 10.1038/s41598-019-49762-1

**Published:** 2019-09-16

**Authors:** Liang Feng, Langfeng Zhou, Xinlei Sun, Qiang Xu, Ping Chen, Xiaolei Wang, Weiwei Liu

**Affiliations:** Institute of Modern Optics, Nankai University, Tianjin, 300350 China

**Keywords:** Super-resolution microscopy, Super-resolution microscopy, Biophotonics, Biophotonics

## Abstract

We present and demonstrate an efficient method for the reconstruction of profiles acquired by multifocal structured illumination microscopy (MSIM) utilizing few raw images. Firstly, we propose a method to produce nine raw multifocal images with enhanced modulation depth to accomplish the uniform illumination of the sample. Then, combing with the parameter of the arrays, we perform the standard construct reconstruction procedure of structured illumination microscopy (SIM) row by row and column by column. Finally, we combine these restored images together to obtain the final image with enhanced resolution and good contrast. Based on theoretical analysis and numerical simulations, this method shows great potential in the field of the image reconstruction of MSIM data.

## Introduction

The wave nature of visible light has hampered the development of optical microscopy in spatial resolution. The Abbe diffraction limit imposes a critical limit on the spatial resolution of optical microscopy, meaning that the maximum available resolution under a wide-field microscope is approximately equal to one-half of the fluorophores emission wavelength which remains two orders of magnitude above the size regime relevant to many molecular reactions and subcellular structures^1^. Therefore, it severely hinders the observation of the fine structure of biological samples. To circumvent the Abbe limit and achieve super-resolution, the past two decades have witnessed the impressive development in super resolution microscopy filed, and a variety of related techniques have come into being^2–5^ to enable measurements within spatial regimes that were previously inaccessible, such as photo-activated localization microscopy (PALM)^6–8^, stochastic optical reconstruction microscopy (STORM)^9–11^, stimulated emission depletion microscopy (STED)^12–14^, and structured illumination microscopy (SIM)^15–18^.

As one of the mainstream super resolution microscopies, SIM applies spatially patterned illumination to elicit sample fluorescence. By frequency-mixing between the excitation pattern and fluorophore density, it visualizes high-resolution information which is inaccessible in the conventional microscope. Linear SIM can double the resolution limit of the conventional imaging system while nonlinear structured light technology can improve the performance of lateral resolution further^17,19,20^. In addition, conventional SIM with sinusoidal illumination patterns offers the advantage which does not require additional complex sample preparation effort. However, under the circumstances of high scattering media and high light intensity, the advantages of SIM based on wide-field imaging are not obvious enough due to the optical aberrations and scattering of the sample. Utilizing point scanning to acquire images, the contrast and quality of the data can be proper maintained while it may be far too slow for monitoring many live cellular processes.

MSIM, with a sparse lattice of excitation foci would dramatically increase acquisition speed and show excellent performance. MSIM could not be well achieved without proper reconstruction methods for the data profiles. The number of original images used for image reconstruction is closely related to the efficiency of the method. The method reported in ref.^21^ is a joint Richardson-Lucy deconvolution algorithm to achieve the reconstruction of MSIM data along with enhanced resolution and good quality. However, the method needs too many images (143 images) and iterations (between 5–30 times) to accomplish the reconstruction, which is complicated and blocks its extensive application. Meanwhile, the method reported in ref.^22^ deconvolves the multifocal-excited, pinholed, scaled and summed (MPSS) images to achieve resolution doubling in live and multicellular organisms. In this method, to fully illuminate the sample, 120 raw multifocal images are acquired and processed to obtain a super resolution image. Although the final images show excellent quality, procedure of data process should been well taken into consideration and might consume lots of effort and time. In this paper, for the sake of image reconstruction of MSIM data, in first step we proposed a method to generate nine raw multifocal images with enhanced modulation depth, then combing with the parameter of the arrays we performed the standard construct reconstruction procedure of SIM row by row and column by column in the these images, respectively. Finally, we combined these restored images to obtain the final image with enhanced resolution and good contrast. Our method holds the merits of high efficiency and flexibility, which has great potential in the field of the reconstruction of MSIM data.

## Result

As a validation of principle, we created artificial MSIM data based on the standard wide-field model and processed it with the proposed reconstruction algorithm which we implemented in MATLAB. The simulations were operated on the resolution test target and the Leaf image, respectively.

### Generation of the multifocal arrays

**Figure 1:**
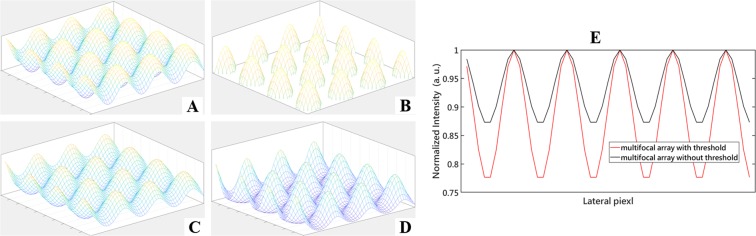
The generation of the multifocal array. (**A**) Result of superimposing the two orthogonal sinusoidal stripes. (**B**) The multifocal array parts with the intensity above the set threshold. (**C**) The wide-field image when A restricted by the OTF of the microscopy system. (**D**) The wide-field image when B restricted by the OTF of the microscopy system. (**E**) Normalized intensity curves along vertical or lateral direction in (**C**,**D**) respectively. As shown in (**E**) the modulation depth of (**D**) may be slighter higher than that of (**C**).

To generate the multifocal array, we superimpose two sinusoidal stripes but orthogonal to each other as shown in Fig. 1(A), of which all have the same parameters including amplitude, frequency. When imaging thick biological tissue, light scattering and depth-induced spherical aberration can significantly weaken the modulation contrast of the structured pattern, which in turn contributes to the restored images with poor signal-to-ratio (SNR)^23^. To maintain the modulation depth of the multifocal array, we set a threshold based on the maximum intensity of the array and only the parts with the amplitude above the threshold of the array is retained. As illustrated in Fig. 1(E), we can see that the modulation depth of multifocal array with threshold is higher than that without the threshold. So far, a preliminary model shown in Fig. 1(B) of the multifocal array has been established. By changing the phase of the two stripes in the X and Y directions, the movement of the multi-focus array can be realized to achieve the entire illumination of the sample.

### The improvement of spatial resolution provided by the proposed method

**Figure 2:**
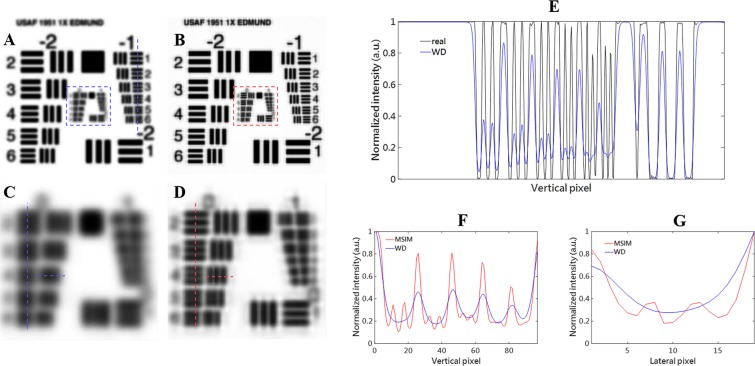
Simulated reconstruction results of the resolution target. (**A**) The wide-field image of the resolution target. (**B**) The reconstructed super-resolved MSIM image of the resolution target. (**C**,**D**) Are the magnifications of the dotted areas in the (**A**,**B**) respectively. (**E**) Normalized intensity curves of the profiles along the dotted vertical lines in (**A**) and the real image. (**F**) Normalized intensity curves of the profiles along the dotted vertical lines in (**C**,**D**) respectively. (**G**) Normalized intensity curves of the profiles along the dotted lateral lines in (**C**,**D**) respectively.

To confirm the improvement of spatial resolution provided by the proposed method, we analyze and compare the simulation results, where the sample is the resolution test target (USAF 1951 1X, Edmund). Figure 2(C,D) are the magnification of respective regions boxed in Fig. 2(A,B), while Fig. 2(A,B) show the wide-field and MSIM images, respectively. As shown in Fig. 2(E), the resolution of the wide-field image is confined to the element 4 of group −1 (around 10 pixels). From Fig. 2(F,G), we find that the reconstructed image can clearly distinguish the element 4 of group 0 (around 5 pixels), achieving a totally twofold resolution enhancement.

### The necessity of setting thresholds to generate the multifocal arrays

**Figure 3:**
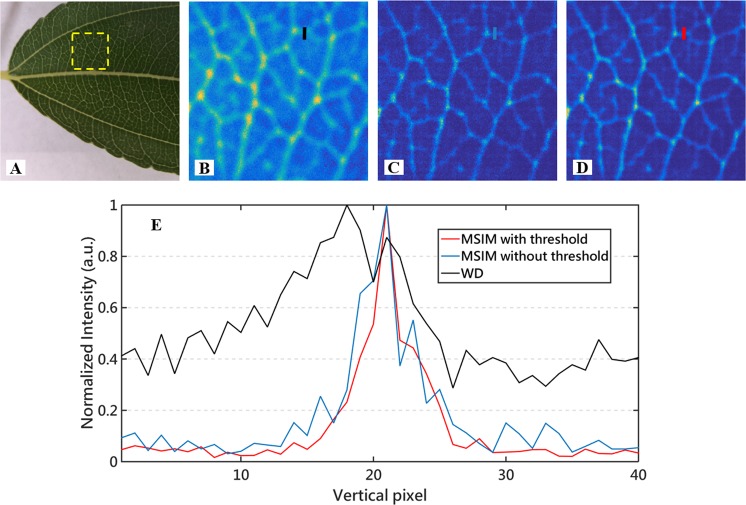
Simulated reconstruction results of the Leaf image under noise circumstance. (**A**) the Leaf photograph, where the area inside the dotted box is the image for subsequent simulations; (**B**) the wide-field image of the area inside the dotted box in (**A**,**C**) the reconstructed image with the nine raw multifocal images corrupted by noise, where the multifocal arrays are generated by superimposing two orthogonal stripes directly; (**D**) the reconstructed image with the nine raw multifocal images corrupted by noise, where the multifocal arrays are generated with our proposed approach (superimposing two orthogonal stripes and retaining the parts above a certain threshold). (**E**) Plots of normalized intensity along the corresponding colored lines in (**B**–**D**).

To illustrate the necessity of setting thresholds to generate the multifocal arrays, we analyzed the results restored by our methods where the raw images are all contaminated by a combination of Poisson noise^24^ and Gaussian noise, which may be more close to the noise in practical experiments. Figure 3(C,D) are the leaf image reconstructed using the multifocal images without threshold and with threshold, respectively, while Fig. 3(B) is the wide-field image. As presented in Fig. 3(E), there is no significant difference between Fig. 3(C,D) in terms of resolution improvement, and the reconstructed image with the multifocal images with the threshold as shown in Fig. 3(D) is slightly better than that image without the threshold shown in Fig. 3(C). The threshold setting to the multifocal arrays can enhance the modulation contrast so as to contribute to the restored image with better contrast and SNR.

## Discussion and Conclusion

With our method, a super resolution image can be obtained just with nine original multifocal images. Compared with methods related to reconstruction of MSIM data^21,22^, the number of raw multifocal images for reconstruction is rather fewer and procedure of the reconstruction is relatively convenient to implement and operate, which is vital and suitable for the live cell imaging and monitoring along with less phototoxicity.

Based on wide-field microscopy, the field of view in conventional SIM is nearly equivalent to that in wide-field microscopy. Spot-scanning SIM can achieve same spatial resolution improvement as the traditional SIM, while its field of view is smaller than that of traditional SIM owning to the limitation of scanning angle of scanning device. If the scanning angle of scanning device is quite large, there would be large aberrations and distortions at the edges of the field of view resulting in degradation of resolution. Parallelization of spot-scanning SIM, MSIM could dramatically improve the efficiency of the data acquisition and realize the same resolution improvement as conventional SIM. Precise knowledge of the excitation pattern is quite imperative to MSIM in both applying digital pinholes and in image reconstruction. These effects are particularly apparent when it comes to imaging highly scattering or thick tissue samples, where the aberrations and noise produce reconstruction artefacts in the final image^25^. Besides, to achieve the fullest resolution improvement, the frequency of the multifocal arrays should be close to the cutoff frequency of the microscopy system.

To achieve the entire illumination of the sample, successive application of nine different patterns can be realized via a single programmable spatial light modulator (SLM) with the merits of program flexibility and the moveable parts is not required. Owing to the characteristics of multifocal arrays, every single excitation focus may have a relatively high intensity and the obtained multifocal arrays might physically reject out-of-focus light and permit imaging the sample even with high scatter property. It should also be taken into consideration that the proposed approach could be applied in two-photon excitation and adaptive optics (AO) for imaging thicker samples with the proper improvement of implements.

In conclusion, we proposed and demonstrated a strategy for the reconstruction of MSIM data. Meanwhile, we presented the feasible approach for the generation of multifocal arrays with considerable modulation depth which is used to maintain the final image with enhanced image contrast. With nine raw multifocal images, the proposed strategy to restore super-resolution images is of high efficiency and has great potential in live cell imaging.

## Methods

### Conventional SIM

In fluorescence microscopy, the illumination pattern *I*_*em*_(*x*) multiplies the sample fluorescence *S*(*x*), consequently eliciting fluorescence emission, which is blurred by diffraction, yielding the image *I*_*ex*_(*x*):

1 $${I}_{em}(x)=({I}_{ex}(x)S(x))\otimes PS{F}_{em}(x)$$

where *PSF*_*em*_(*x*) is the emission point spread function (blurred image of a point source), and ⊗ denotes the convolution operation. Fourier transformation of Eq. (1) yields

2 $${\tilde{I}}_{em}(k)=({\tilde{I}}_{ex}(k)\tilde{S}(k))\cdot OT{F}_{em}(k)$$

where the tilde represents the Fourier transform of the function and optical transfer function *OTF*_*em*_(*k*) is the Fourier transform of the *PSF*_*em*_(*x*).

As for conventional SIM, the illumination can be approximately described as:

3 $${I}_{ex}(x)={I}_{0}\cdot [1+m\cdot \,\cos \,(2\pi {k}_{0}x+{\phi })]$$

where *k*_0_ and *φ* are the spatial frequency and the phase of the illumination patterns, and *I*_0_ is the peak illumination intensity. By Fourier transforming Eq. (3) and substituting into Eq. (2), the expression for the observed image can be obtained as:

4 $${\tilde{I}}_{em}(k)={I}_{0}\cdot [\tilde{S}(k)+\frac{m}{2}\tilde{S}(k+{k}_{0}){e}^{-i{\phi }}+\frac{m}{2}\tilde{S}(k-{k}_{0}){e}^{i{\phi }}]\cdot OT{F}_{em}(k)$$

The first term in Eq. (4) denotes all the spatial frequencies normally observed by the microscope while the two remaining terms gives rise to resolution enhancement as they modulate high frequency components into the passband of the *OTF*_*em*_(*k*). By obtaining three images with three different phases (*φ*_*x*,*y*_) of the illumination pattern we can obtain a system of three independent linear equations, allowing us to extract the three terms in Eq. (4). After that, we untangle and restore these frequency components to their proper location in Fourier space to reconstruct a super-resolved image. The maximum frequency of the illumination pattern is subject to the *OTF*_*em*_(*k*), as same as in the conventional wide-field microscopy. Consequently, it is possible to increase the resolution of the optical microscope along the pattern’s direction by a factor of approximately two in this case.

### Method for the construction of MSIM

**Figure 4:**
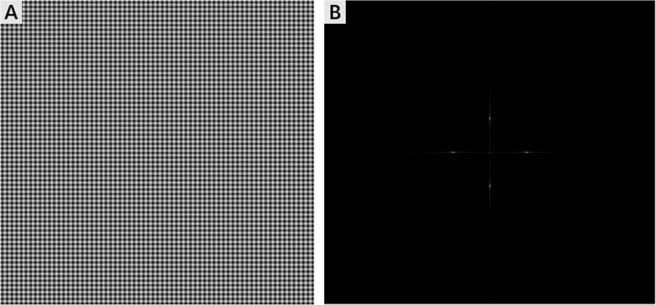
Multifocal array and its Fourier space. (**A**) The multifocal array. (**B**) Corresponding Fourier space of the array.

As for MSIM, the illumination shown in Fig. 4(A) can be approximately described as:

5 $${I}_{ex}(x,y)={I}_{0}\cdot [1+m\cdot \,\cos \,(2\pi {k}_{x}x+{{\phi }}_{x})+m\cdot \,\cos \,(2\pi {k}_{y}y+{{\phi }}_{y})]$$

where *k*_*x*,*y*_ and *φ*_*x*,*y*_ are the spatial frequency and the phase of the two illumination patterns along the X or Y direction, respectively. Substituting the Fourier transformation of Eq. (5) into Eq. (2) yields

6 $${\mathop{I}\limits^{ \sim }}_{em}(k)={I}_{0}\cdot [\begin{array}{c}\mathop{S}\limits^{ \sim }(k)+\frac{m}{2}\mathop{S}\limits^{ \sim }(k+{k}_{x})\cdot {e}^{-i{\phi }_{x}}+\frac{m}{2}\mathop{S}\limits^{ \sim }(k-{k}_{x})\cdot {e}^{i{\phi }_{x}}\\ +\frac{m}{2}\mathop{S}\limits^{ \sim }(k+{k}_{y})\cdot {e}^{-i{\phi }_{y}}+\frac{m}{2}\mathop{S}\limits^{ \sim }(k-{k}_{y})\cdot {e}^{i{\phi }_{y}}\end{array}]\cdot OT{F}_{em}(k)$$

Compared with the Eq. (4), the Eq. (6) has five terms indicating that it has frequency shift along X and Y directions. In this case, the MSIM can enhance the spatial resolution of the sample in two directions simultaneously.

To construct the super-resolved images, we make it by performing the reconstruction from two directions separately based on standard reconstruction procedure of SIM. The multifocal arrays we generated here have frequency distributions in the X and Y direction, for the reason that we superimposed two sinusoidal stripes that are orthogonal to each other. After performing reconstruction with the raw multifocal images, we can obtain the final images with isotropic resolution improvement along the two directions simultaneously. To extract the three frequency terms in the X direction, we should shift the horizontal stripes three times to obtain a system of three independent equations. Meanwhile, to extract the three frequency terms in the Y direction, we should shift the vertical stripes three times to obtain a system of three independent equations. Therefore, nine raw multifocal images are required, which can accomplish the uniform illumination of the sample as well.

**Figure 5:**
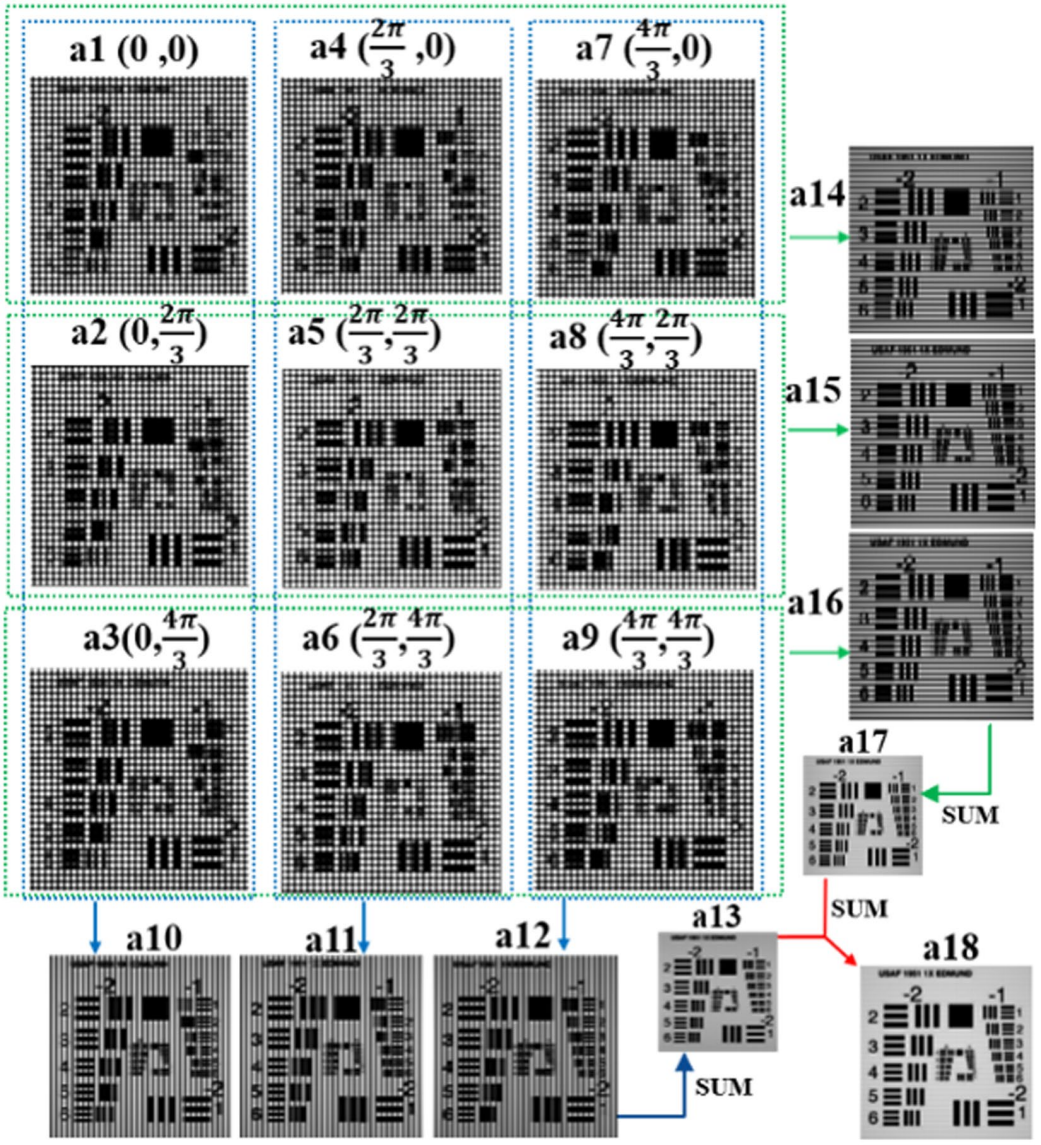
The procedure of the reconstruction for MSIM data. (**a1**–**a9**) The nine raw images. The bracket on their side shows the phases of the image in X and Y directions, respectively. (**a10**–**a12**) Are the reconstructed images by column while (**a14**–**a16**) are the reconstructed images by row. (**a13**) Is the result by superimposing (**a10**–**a12**) while (**a17**) is the result by superimposing (**a14**–**a16**). (**a18**) The final reconstructed image with spatial resolution enhanced in the X and Y directions.

To obtain the super-resolved images, the restore procedure is performed in in the two direction respectively, after that the reconstruction results are superimposed. Nine images are arranged artificially in three rows and three columns, according to their phase relationship of each of the multifocal images in two directions: three multifocal array images in the same column have the same phase in the X direction and a phase difference of 2*π*/3 in the X direction, while three multifocal array images in the same column have the same phase in the X direction and a phase difference of 2*π*/3 in the Y direction, as shown in Fig. 5(a1–a9), respectively.

**Figure 6:**
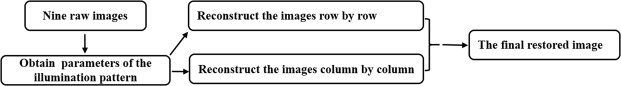
Schematic of the reconstruction procedure for MSIM data.

**Figure 7:**
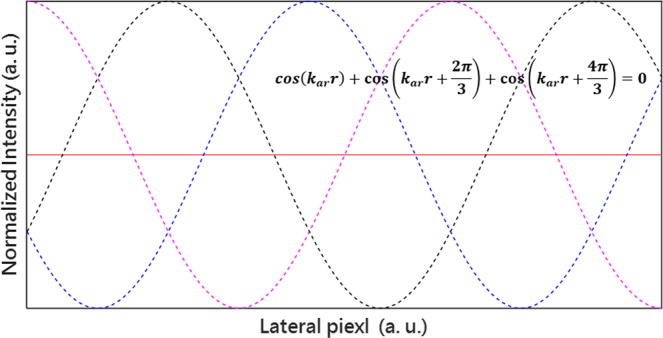
Superimposing the three obtained images to eliminate influence of the stripes on the quality of the image. The three dotted curves in the figure correspond to three terms in the formula respectively, where *k*_*ar*_ represents the arbitrary spatial frequency. As the figure and formula illustrated, only the direct term irrelative with the frequency as the red line shown is remaining after superimposing the three obtained images, leading to no strips in the final image.

Figure 6 illustrates the schematic of the reconstruction procedure for MSIM data. We first estimate the parameters of the illumination patterns and then reconstruct the images row by row and column by column, finally we add up these restored images together to obtain the final image with enhancement of spatial resolution in the X and Y directions. The specific reconstruction procedures are described in detail as follows:

1. Applying three-phase standard reconstruction procedure column by column, we get three restored images, as shown in Fig. 5(a10–a12). Compared with the wide-field images, the resolution of these three images is enhanced in the Y direction.
  Since the reconstruction process in the X direction is not involved yet, the three obtained images still have a strip with a same frequency and have a phase difference of 2*π*/3 between each other. As Fig. 7 illustrated, we superimpose them together to eliminate influence of the stripes on the quality of the image and acquire an image, as demonstrated in Fig. 5(a13), with enhanced resolution in the X direction.
2. Repeat the step (1) by row by row, and obtain the image Fig. 5(a17) with enhanced resolution in the Y direction.
3. Add up the obtained images, Fig. 5(a13,a17) in the two previous steps, and the final image Fig. 5(a18) with improved resolution in both directions can be obtained.

The spatial frequency of the multifocal array is limited by the *OTF*_*em*_(*k*) of the optical system as well. Compared with the traditional fluorescence microscopy, the proposed method could improve the spatial resolution by a factor of two as the traditional SIM under the linear situation.

## Data Availability

The datasets generated and analyzed during the current study are available from the corresponding author on reasonable request.
